# First-in-Human Phase I/IIa Study of the First-in-Class CDK2/4/6 Inhibitor PF-06873600 Alone or with Endocrine Therapy in Patients with Breast Cancer

**DOI:** 10.1158/1078-0432.CCR-24-2740

**Published:** 2025-04-17

**Authors:** Timothy A. Yap, Jonathan W. Goldman, Shaveta Vinayak, Antoaneta Tomova, Erika Hamilton, Yoichi Naito, Antonio Giordano, Igor Bondarenko, Toshinari Yamashita, Li Zhou, Allison Moreau, Heather Neumann, Jessica Tougias, Feng Liu, Jennifer Park, Maria Delioukina, Komal Jhaveri

**Affiliations:** 1 Department of Investigational Cancer Therapeutics, The University of Texas MD Anderson Cancer Center, Houston, Texas.; 2 UCLA Hematology/Oncology, Los Angeles, California.; 3 University of Washington/Fred Hutchinson Cancer Center, Seattle, Washington.; 4 Complex Oncology Center Plovdiv, Plovdiv, Bulgaria.; 5 Sarah Cannon Research Institute, Nashville, Tennessee.; 6 National Cancer Center Hospital East, Chiba, Japan.; 7 Dana-Farber Cancer Institute, Harvard Medical School, Boston, Massachusetts.; 8 Municipal Non-profit Enterprise “City Clinical Hospital #4” of Dnipro City Council, Dnipro, Ukraine.; 9 Kanagawa Cancer Center, Kanagawa, Japan.; 10 Pfizer Inc., La Jolla, California.; 11 Pfizer Inc., Redding, Connecticut.; 12 Memorial Sloan Kettering Cancer Center, New York, New York.; 13 Weill Cornell Medical College, New York, New York.

## Abstract

**Purpose:**

The discovery that cyclin E overexpression is a key cyclin-dependent kinase (CDK) 4/6 inhibitor resistance mechanism has reinvigorated interest in targeting CDK2 and the simultaneous inhibition of CDK2/4/6 as a novel therapeutic approach. This first-in-human study assessed the safety, tolerability, pharmacokinetics, pharmacodynamics, and efficacy of PF-06873600, the first-in-class inhibitor of CDK2/4/6.

**Patients and Methods:**

Dose escalation included 78 patients with advanced breast cancer, triple-negative breast cancer, or ovarian cancer who received oral PF-06873600 at doses ranging from 1 to 50 mg twice daily (part 1A, *n* = 51) or PF-06873600 with endocrine therapy (part 1B, *n* = 16; part 1C, *n* = 11) to determine the recommended dose for expansion (RDE). Dose expansion (part 2A, *n* = 45; part 2C, *n* = 28) assessed preliminary antitumor activity, safety, and tolerability at the RDE in combination with fulvestrant in patients with hormone receptor^+^/HER2^−^ metastatic breast cancer. Pharmacodynamics and translational readouts were assessed by measuring phosphorylated Rb and Ki67 in tumor biopsies and ctDNA.

**Results:**

The RDE of PF-06873600 was 25 mg twice daily. During dose escalation, 6 of 42 (14.3%) evaluable patients had treatment-related dose-limiting toxicities. The most common all-causality adverse events (*N* = 151) were nausea (62.9%), anemia (44.4%), and fatigue (43.7%). Reductions in Ki67-positive cells, phosphorylated Rb histo-score, and ctDNA levels were observed. Three RECIST partial responses (PR) were observed in part 1. In part 2A, there were three PRs (objective response rate, 6.7%; 95% confidence interval, 1.4%–18.3%), and in part 2C, there were five PRs (objective response rate, 22.7%; 95% confidence interval, 7.8%–45.4%).

**Conclusions:**

PF-06873600 demonstrated a benefit–risk profile consistent with the CDK4/6 inhibitor class of drugs, with preliminary clinical activity in hormone receptor^+^/HER2^−^ metastatic breast cancer.

**Translational Relevance**

The development of resistance to cyclin-dependent kinase (CDK) 4/6 inhibitors (CDK4/6i) in hormone receptor^+^/HER2^−^ metastatic breast cancer is common, and second-line therapy options are limited following failure on CDK4/6i. Understanding the mechanisms of resistance is an important area of focus. Research has shown that cyclin E activation of CDK2 can drive the cell cycle independently of CDK4 and CDK6, and CDK4/6-inhibited cells can activate CDK2. Hence, simultaneous inhibition of CDK2/4/6 may offer a novel therapeutic approach to prolong clinical benefit. The current study investigated the safety, tolerability, pharmacokinetics, pharmacodynamics, and efficacy of the novel first-in-class CDK2/4/6i PF-06873600 in patients with advanced breast cancer. A benefit–risk profile consistent with the CDK4/6i class of drugs was observed, with preliminary clinical activity observed in hormone receptor^+^/HER2^−^ metastatic breast cancer.

## Introduction

Cyclin-dependent kinases (CDK) and cyclins play a key role in driving cell-cycle transitions and cell division (1, 2). Dysregulation of the cell-cycle machinery is a major hallmark of cancer, which can result in overactivation of CDKs and uncontrolled cell proliferation. Although the FDA approvals of the CDK4/6 inhibitors (CDK4/6i) palbociclib, ribociclib, and abemaciclib have transformed the treatment of metastatic hormone receptor-positive (HR^+^) and HER2-negative (HER2^−^) breast cancer, they remain a noncurative treatment option, as the development of resistance is common and nearly inevitable (3–5). Hence, there is an unmet clinical need for an improvement in treatment approaches to positively affect patients.

Abnormal CDK2/cyclin E1 activation due to *CCNE1* gene overexpression is a key resistance mechanism to CDK4/6 inhibition. Cyclin E activates CDK2, which can drive the cell cycle independently of CDK4 and CDK6 (6), and data support the role of high cyclin E1 in resistance to CDK4/6 inhibition. In preclinical models, resistance to CDK4/6 inhibition can be overcome by concomitant inhibition of CDK2. For example, palbociclib-resistant breast cancer (MCF-7pR) cells that had undergone prolonged treatment with palbociclib acquired *CCNE1* gene amplification and sustained high levels of CDK2 Thr160 phosphorylation, and the silencing of *CCNE1* or *CDK2* alone in these cells did not affect cell-cycle arrest. However, when silenced in combination with palbociclib, cell-cycle arrest increased with a reduction in cell growth (5). Hence, cells that had acquired resistance to CDK4/6 inhibition due to adaptive CDK2 activation can be resensitized by cotargeting CDK2.

The simultaneous inhibition of CDK2/4/6 may provide a new therapeutic strategy to prolong clinical benefit. The association of amplification and/or overexpression of cyclins E1 and E2 with CDK4/6i resistance has renewed interest in developing CDK2 inhibitors, which may overcome CDK4/6i resistance, whether intrinsic or acquired. This prompted the development of PF-06873600, the first-in-class selective inhibitor of CDK2/4/6, which has demonstrated antitumor activity as a single agent and in combination with endocrine therapy (ET) in multiple *in vivo* tumor models (7, 8).

This was the first-in-human clinical study to evaluate the safety, pharmacokinetics (PK), pharmacodynamics, and preliminary antitumor activity of PF-06873600, alone and in combination with ET, in patients with HR^+^/HER2^−^ advanced breast cancer or metastatic breast cancer (mBC) who were CDK4/6i-naïve but had prior ET or had failed prior combination CDK4/6i and ET. Additionally, during dose escalation, PF-06873600 was investigated in tumor types that have the potential for increased cyclin E expression and/or CDK2 activity, including locally recurrent/advanced or metastatic triple-negative breast cancer (TNBC) and advanced platinum-resistant ovarian cancer.

## Patients and Methods

### Study design and endpoints

**Figure 1.**
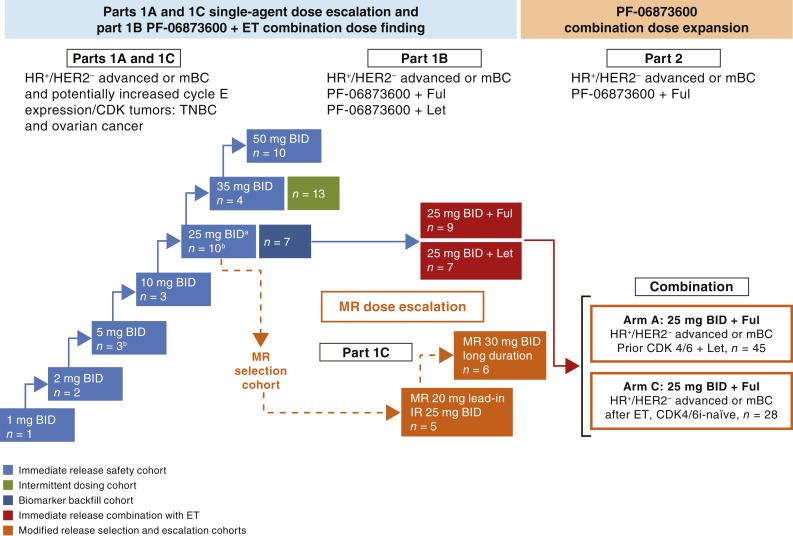
Overall study schema. ^a^25 mg twice daily (BID) was the RDE. ^b^One patient was untreated. AI, aromatase inhibitor; CDK, cyclin-dependent kinases; CDK4/6i, CDK4/6 inhibitor; ET, endocrine therapy; Ful, fulvestrant; HER2^−^, human epidermal growth receptor 2-negative; HR^+^, hormone receptor-positive; IR, immediate release; Let, letrozole; mBC, metastatic breast cancer; MR, modified release; RDE, recommended dose for expansion; TNBC, triple negative breast cancer.

This was a first-in-human phase I/IIa, open-label, multicenter, nonrandomized, multiple-dose, safety, tolerability, pharmacokinetic, and pharmacodynamic study of PF-06873600 administered as a single agent in sequential dose levels and then in combination with ET, which was conducted in accordance with CONSORT guidelines (ClinicalTrials.gov identifier: NCT03519178). The overall study design is depicted in Fig. 1.

Part 1 included adult patients with HR^+^/HER2^−^ advanced breast cancer or mBC (third- or fourth-line setting), locally recurrent/advanced or metastatic TNBC, or advanced platinum-resistant epithelial ovarian cancer/fallopian tube cancer/primary peritoneal cancer who received oral PF-06873600 at doses of 1 to 50 mg twice daily or oral PF-06873600 plus ET (letrozole or fulvestrant). Primary endpoints for part 1 dose escalation determined safety and tolerability to define dose-limiting toxicities (DLT), estimate the maximum tolerated dose, and select the recommended dose for expansion (RDE). Safety endpoints included adverse events (AE) characterized by type, frequency, severity [as graded by NCI Common Terminology Criteria for Adverse Events (CTCAE) version 4.03], timing, seriousness, and relationship to study therapy. Laboratory abnormalities were characterized by type, frequency, severity (as graded by NCI CTCAE version 4.03), timing, vital sign abnormalities, and heart rate–corrected QT interval (e.g., QTcF). Secondary endpoints included PK, preliminary antitumor activity as assessed using RECIST version 1.1, time-to-event endpoints including objective response rate (ORR), duration of response (DoR), progression-free survival (PFS), time to progression (TTP), and modulation of tumor CDK biomarkers [phosphorylated Rb (pRb), Ki67]. Exploratory endpoints included molecular profiling of tumor and ctDNA and the relationship to clinical outcome.

Part 2 was a dose expansion, open-label, multicenter, nonrandomized study to assess the preliminary antitumor activity and the safety and tolerability of PF-06873600 at the RDE in combination with fulvestrant. Part 2 included patients with HR^+^/HER2^−^ advanced breast cancer or mBC (second- or third-line setting) with prior CDK4/6 and a nonsteroidal aromatase inhibitor (part 2A) or patients who were CDK4/6i-naïve but had prior ET (part 2C). Primary endpoints included preliminary antitumor activity measures for efficacy as assessed using RECIST version 1.1. Safety endpoints were the same as for part 1. Secondary endpoints included time-to-event endpoints, PK parameters, and modulation of tumor pharmacodynamic (PD) biomarkers (pRb, Ki67). Exploratory endpoints included molecular profiling of tumor and ctDNA and the relationship to clinical outcome.

### Patient eligibility

Patients were aged ≥18 years with a histologically or cytologically proven diagnosis of cancer and measurable disease defined by RECIST version 1.1, Eastern Cooperative Oncology Group (ECOG) performance status (PS) 0 to 1, and adequate organ function.

Part 1 involved patients with HR^+^/HER2^−^ advanced breast cancer or mBC (third- or fourth-line setting) who had received one prior line of combined CDK4/6i and ET therapy and one or two prior lines of cytotoxic chemotherapy in the advanced setting. Patients with TNBC had received up to two prior lines of chemotherapy in the advanced or metastatic setting, and patients with ovarian cancer had received up to three prior lines of therapy.

Part 2 arm A (PF-06873600 combination with fulvestrant) included patients with HR^+^/HER2^−^ locally advanced breast cancer or mBC (second- or third-line setting) who had received one prior line of combined CDK4/6i and a nonsteroidal aromatase inhibitor with disease progression either on or after treatment. One prior line of cytotoxic chemotherapy in the advanced or metastatic setting was allowed. Part 2C included patients with HR^+^/HER2^−^ locally advanced breast cancer or mBC naïve to CDK4/6i who had not received a CDK4/6i as adjuvant therapy or in the advanced or metastatic setting. One prior line of cytotoxic chemotherapy in the advanced or metastatic setting was allowed if the patient was CDK4/6i-naïve. Arm B was designed to involve PF-06873600 in combination with aromatase inhibitors, and Arm C involved PF-06873600 in combination with fulvestrant. Arm B of part 2 was deprioritized by the sponsor, so patients were not enrolled, and there are no results to report.

Patients were not eligible if they had symptomatic central nervous system metastases or other active malignancy within 3 years prior to randomization, except for adequately treated basal cell or squamous cell skin cancer or carcinoma *in situ*. Previous high-dose chemotherapy requiring stem cell rescue was not permitted. Patients who had major surgery or radiotherapy 4 weeks prior to study entry were not included.

The protocol, protocol amendments, informed consent forms, investigator brochure, and other relevant documents were submitted to an Institutional Review Board/Independent Ethics Committee by the investigator and reviewed and approved by the Institutional Review Board/Independent Ethics Committee before the study initiation. This study was conducted in accordance with the protocol and consensus ethical principles derived from international guidelines, including the Declaration of Helsinki and Council for International Organizations of Medical Sciences International Ethical Guidelines, applicable International Council for Harmonisation's Good Clinical Practice Guidelines, applicable International Organization for Standardization 14155 guidelines, medical device guidelines, and other applicable laws and regulations, including privacy laws. All patients provided written informed consent before being enrolled in the study.

### Treatment

In part 1A, dose escalation monotherapy, patients received escalating doses of a single-agent immediate release (IR) formulation of PF-06873600, 1 to 50 mg twice daily orally on a continuous basis, with dose escalation following the modified toxicity probability interval method. Seven dose levels were evaluated: 1, 2, 5, 10, 25, 35, and 50 mg. An alternative intermittent dosing regimen (5 days on/2 days off) was also explored at 35 mg twice daily. Part 1B combination dose-finding involved PF-06873600 IR 25 mg twice daily with ET (letrozole or fulvestrant, each administered per standard of care). Letrozole was administered orally once daily together with PF-06873600. No dose adjustment for letrozole was permitted, but dosing interruptions were allowed. Fulvestrant 500 mg was administered intramuscularly on cycle 1 day 1 and day 15, cycle 2 day 1, and then monthly thereafter (±3 days) to accommodate dosing on day 1 of each cycle. No dose adjustment for fulvestrant was permitted. Part 1C evaluated two modified release (MR) formulations of PF-06873600: one in the single-dose setting and the other in the twice-daily setting. A cycle was defined as 28 days, regardless of missed doses or dose delays.

The single-agent PF-06873600 RDE (25 mg twice daily) from part 1A was used to initiate the part 2 dose expansion arm studies involving combination with fulvestrant administered in 28-day cycles (parts 2A and 2C). As mentioned above, arm B of part 2 was deprioritized by the sponsor, so patients were not enrolled in arm B. Treatment continued until disease progression, uncontrollable toxicity, patient or investigator decision to discontinue, or study termination.

### Safety

AEs were graded by NCI CTCAE version 4.03.

### PK

Plasma samples were analyzed for PF-06873600 concentrations at Q2 Solutions using a validated analytic assay in compliance with Pfizer standard operating procedures. PF-06873600 samples were assayed by a validated LC/MS-MS using positive ionization mode. PK parameters were calculated using noncompartmental analysis of plasma concentration–time data.

### Biomarkers/pharmacodynamics

#### Tumor pharmacodynamic biomarkers

The pharmacodynamic effects of PF-06873600 were assessed by IHC staining of pRb and Ki67 in tumor biopsy samples collected at screening and day 1 of cycle 2 (4 hours after dose). Immunostaining was performed on 4 mm formalin-fixed, paraffin-embedded tissue sections using rabbit monoclonal anti-Rb (Ser807/811) antibody (clone D20B12, Cell Signaling Technology) and rabbit monoclonal anti-Ki67 antibody (Ventana Medical Systems), respectively. pRb and Ki67 expressions were assessed semiquantitatively by a board-certified pathologist. Scoring was performed using the percentage of cells positive for Ki67 or a histo-score (H-score) methodology for pRb based on staining intensity and the percentage of positive cells. Intensity was scored as 0, none; 1, weak; 2, moderate; or 3, strong, and H-scores were calculated as follows: H-score = [fraction of cells with intensity grade 1 (%)] + [fraction of cells with intensity grade 2 (%) × 2] + [fraction of cells with intensity grade 3 (%) × 3].

#### ctDNA molecular response

Plasma samples were collected at cycle 1 day 1, cycle 1 day 15, and the end of treatment (EOT). Genomic alterations in ctDNA were detected using the next-generation sequencing–based 74-gene Guardant360 platform (Guardant Health). ctDNA molecular response between the two samples was determined as described by Mack and colleagues (9). Briefly, after subtracting germline variants and potential clonal hematopoiesis of indeterminate potential, the mean variant allele frequency (meanVAF) of somatic variants (including single-nucleotide variants, insertions and deletions, and fusions) with a mutant molecular count above a proprietary threshold was calculated for each sample by averaging the VAFs of all variants detected among the two samples. The relative change of meanVAF of the posttreatment sample from the baseline sample was then calculated as the ctDNA molecular response. Samples lacking somatic variants with sufficient mutant molecular counts or the absence of any somatic alterations at both time points were considered ctDNA low and not evaluable for molecular response.

#### Antitumor activity

Antitumor activity was assessed through radiological tumor assessments conducted at baseline, during treatment, whenever disease progression was suspected (e.g., symptomatic deterioration), and at the time of withdrawal from treatment (if not done in the previous 8 weeks). Responses were assessed using RECIST version 1.1.

### Statistical analyses

A modified toxicity probability interval method [targeting a DLT rate of 27.5% and an acceptable DLT interval (22.5%–32.5%)] was used to guide dose escalation and monitor the DLTs. DLT was the primary endpoint of the study, summarized by dose level using the per-protocol analysis set for patients in the dose escalation portion of the study. Efficacy analysis was summarized by dose cohorts for part 2 of the study. For part 1, no formal efficacy analysis was presented. For part 2, tumor response was presented in the form of patient data listings that included, but were not limited to, tumor type, starting dose, tumor response at each visit, and best overall response. In addition, progression date, death date, date of first response, last tumor assessment date, and date of last contact were listed. ORR, PFS, overall survival, TTP, and DoR were summarized and presented as data permitted. For response-based metrics (best overall response, ORR, and DoR), analyses were presented on the basis of confirmed responses. Analyses of PFS and TTP were defined as the time from the date of the first dose. Summaries of the number and percentage of patients with PFS and TTP and data listings by tumor type and dose were generated based on data availability. The 95% confidence intervals (CI) for medians and quartiles were generated based on the Brookmeyer–Crowley method, whereas CIs for the estimated probability of an event at a particular time point were generated using the Greenwood formula. DoR was summarized with the number of responders, the number and percentage of events/censorship, mean, standard deviation, and minimum, maximum, and median duration in units of months. ORR was summarized by the number of patients meeting respective criteria as a percentage. The 95% CIs were generated for PFS, TTP, and ORR based on available data.

### Data availability

Upon request, and subject to review, Pfizer will provide the data that support the findings of this study. Subject to certain criteria, conditions, and exceptions, Pfizer may also provide access to the related individual deidentified participant data. See https://www.pfizer.com/science/clinical-trials/trial-data-and-results for more information. Pfizer has partnered with Vivli to share our Pfizer data (https://vivli.org/). Please fill out the Vivli inquiry form at https://vivli.org/members/enquiries-about-studies-not-listed-on-the-vivli-platform/. If a user has further questions about the Vivli process, please contact support@vivli.org. Additional information can also be found on the Pfizer/Vivli member page.

## Results

### Patients

A total of 153 patients were enrolled, and 151 patients were treated (Supplementary Table S1). In part 1A, 53 patients were assigned to treatment, and 51 patients were treated. For parts 1B and 1C, 16 and 11 patients, respectively, were assigned to treatment and treated. A total of 45 and 28 patients were assigned to treatment and treated in parts 2A and 2C, respectively. Of the 151 patients in the safety analysis set, all were female, most (71.5%) were White, and the median age was 59.0 (range: 28–84) years. A total of 81 patients (53.6%) had a baseline ECOG PS of 0, 69 (45.7%) had a baseline ECOG PS of 1, and 1 (0.7%) had a baseline ECOG PS of 2. Of 151 patients in the safety analysis set, 144 (95.4%) had breast cancer [136 (90.1%) HR^+^/HER2^−^, 3 (2.0%) HR^+^/HER2^+^, 5 (3.3%) TNBC]. Seven (4.6%) had ovarian cancer (Table 1). Cohorts 1B, 2A, and 2C included patients with mBC only.

**Table 1.** Demographics, baseline characteristics, and prior treatments—safety analysis set (*N* = 151).

	Part 1 + part 2 (*N* = 151)
Age, median (range), in years	59.0 (28–84)
Gender, *n* (%)	
Female	151 (100)
Race, *n* (%)	
White	108 (71.5)
Black or African American	10 (6.6)
Asian	22 (14.6)
Native Hawaiian or Other Pacific Islander	1 (0.7)
Not reported	10 (6.6)
Tumor type, *n* (%)	
Breast cancer	144 (95.4)
HR^+^/HER2^+^	3 (2.0)
HR^+^/HER2^−^ (mBC)	136 (90.1)
Triple-negative	5 (3.3)
Ovarian cancer	7 (4.6)
Baseline ECOG PS, *n* (%)	
0	81 (53.6)
1	69 (45.7)
2	1 (0.7)
Prior anticancer surgery, *n* (%)	103 (68.2)
Prior radiotherapy, *n* (%)	58 (38.4)
Prior adjuvant/neoadjuvant therapy, *n* (%)	106 (70.2)
Number of prior systemic lines of therapy in advanced/metastatic setting, median (range)	3.0 (0–8)
Number of prior lines of ET in advanced/metastatic setting, median (range)	2.0 (0–6)
Number of prior lines of chemotherapy in advanced/metastatic setting, median (range)	1.0 (0–4)
Any prior ET in advanced/metastatic setting, *n* (%)	110 (72.8)
Any prior CDK4/6i, *n* (%)	104 (68.9)
Any prior chemotherapy, *n* (%)	98 (64.9)
Any prior immunotherapy/biologics, *n* (%)	15 (9.9)

Abbreviations: ECOG, Eastern Cooperative Oncology Group; HER, human epidermal growth factor receptor; HR, hormone receptor; mBC, metastatic breast cancer; PS, performance status.

At the primary completion date, seven patients (4.6%) were ongoing; most (95.4%) discontinued PF-06873600 treatment, including all patients in parts 1A, 1B, and 1C; 44 of 45 (97.8%) in part 2A; and 22 of 28 (78.6%) in part 2C. The main reasons for discontinuation from PF-06873600 treatment were progressive disease (52.9%), withdrawal by the patient (9.8%), and global deterioration of health status (7.8%).

### Safety

The median duration of PF-06873600 treatment was as follows: part 1A: 58.0 days, range 5 to 1,093 days; part 1B: 125.5 days, range 11 to 419 days; part 1C: 112.0 days, range 25 to 466 days; part 2A: 120.0 days, range 18 to 636 days; and part 2C: 148.5 days, range 1 to 413 days.

All-causality treatment-emergent adverse events (TEAE) were reported in 147 of 151 patients (97.4%; parts 1 and 2). The most frequently reported (≥20% of patients) were nausea (62.9%), anemia (44.4%), fatigue (43.7%), neutropenia (37.7%), vomiting (35.8%), headache (29.1%), alopecia (28.5%), leukopenia (25.8%), constipation (25.2%), and diarrhea (22.5%). A total of 69 patients (45.7%) had grade 3 and grade 4 all-causality TEAEs (grade 3, 34.4%; grade 4, 11.3%).

Treatment-related TEAEs were reported in 138 of 151 patients (91.4%; parts 1 and 2; Table 2). The most frequently reported (≥20% of patients) were nausea (60.9%), anemia (41.1%), fatigue (39.7%), neutropenia (37.1%), vomiting (33.8%), alopecia (27.8%), leukopenia (24.5%), and headache (21.2%). Fifty-four patients (35.8%) had grade 3 and grade 4 treatment-related TEAEs (grade 3, 25.8%; grade 4, 9.9%). A total of 34 patients (22.5%) had all-causality serious adverse events (SAE). The most frequently reported were febrile neutropenia [five patients (3.3%)] and abdominal pain, colitis, and hypotension [each in three patients (2.0%)]. Thirteen patients (8.6%) had treatment-related SAEs, the most frequently reported being febrile neutropenia [five patients (3.3%)] followed by neutropenia, colitis, and stomatitis [each in two patients (1.3%)]. A total of two (1.3%) patients reported treatment-related grade 5 TEAEs [cardiac arrest and gastrointestinal (GI) bacterial infection were each reported in one patient]. The patient with cardiac arrest (part 1A, PF-06873600 50 mg twice daily) had grade 4 febrile neutropenia and thrombocytopenia, followed by multiorgan dysfunction syndrome leading to death. The patient with a GI bacterial infection (part 2C, PF-06873600 25 mg twice daily IR/fulvestrant) had an ongoing grade 4 decreased white blood cell count AE.

**Table 2.** Summary of TEAEs (>15%) by preferred term and maximum CTCAE grade in descending frequency order (treatment-related, all cycles)—safety analysis set parts 1 and 2 (*N* = 151).

Preferred term	Grade 1	Grade 2	Grade 3/4	Grade 5	Total
Any AE	27 (17.9)	55 (36.4)	54 (35.8)	2 (1.3)	138 (91.4)
Nausea	65 (43.0)	26 (17.2)	1 (0.7)	0	92 (60.9)
Anemia	13 (8.6)	28 (18.5)	21 (13.9)	0	62 (41.1)
Fatigue	23 (15.2)	25 (16.6)	12 (7.9)	0	60 (39.7)
Neutropenia	6 (4.0)	19 (12.6)	31 (20.5)	0	56 (37.1)
Vomiting	38 (25.2)	12 (7.9)	1 (0.7)	0	51 (33.8)
Alopecia	21 (13.9)	21 (13.9)	0	0	42 (27.8)
Leukopenia	5 (3.3)	23 (15.2)	9 (6.0)	0	37 (24.5)
Headache	28 (18.5)	4 (2.6)	0	0	32 (21.2)
Thrombocytopenia	16 (10.6)	4 (2.6)	9 (6.0)	0	29 (19.2)
Diarrhea	21 (13.9)	4 (2.6)	2 (1.3)	0	27 (17.9)
Constipation	13 (8.6)	10 (6.6)	0	0	23 (15.2)

All values are *n* (%).

Abbreviation: CTCAE, Common Terminology Criteria for Adverse Events.

Treatment-related TEAEs were reported in 45 of 45 patients (100%) in part 2A and 24 of 28 patients (85.7%) in part 2C (Supplementary Table S2).

A total of 78 patients (51.7%) had TEAEs leading to dose interruptions. The most frequently reported TEAEs leading to dose interruptions were neutropenia in 27 patients (17.9%) and anemia and nausea each in 17 patients (11.3%). A total of 32 patients (21.2%) had TEAEs leading to dose reductions. The most frequently reported were neutropenia in nine patients (6.0%), fatigue in eight patients (5.3%), and nausea in seven patients (4.6%). A total of 11 patients (7.3%) had TEAEs leading to discontinuation.

In part 1A, 42 patients were evaluable for DLT, and six (14.3%) had DLTs all considered treatment-related—PF-06873600 35 mg: grade 4 neutrophil count decreased (*n* = 1) and grade 4 neutrophil count decreased and grade 4 platelet count decreased (*n* = 1) and PF-06873600 50 mg twice daily: grade 4 febrile neutropenia (*n* = 1); grade 3 fatigue (*n* = 1); grade 4 febrile neutropenia, grade 4 thrombocytopenia, grade 5 cardiac arrest, and grade 4 multiple organ dysfunction syndrome (*n* = 1); and grade 3 febrile neutropenia and grade 3 colitis (*n* = 1). The RDE was determined to be PF-06873600 25 mg twice daily in the single-agent and combination dosing groups. No DLTs were reported at the monotherapy RDE in the single-agent or combination groups (Table 3).

**Table 3.** Summary of DLT by system organ class and preferred term (all causalities). DLT—evaluable analysis set.

Number (%) of patients by system organ class and preferred term	35 mg twice daily (*N* = 2), *n* (%)	50 mg twice daily (*N* = 8), *n* (%)
Patients with any DLTs	2 (100.0)	4 (50.0)
Febrile neutropenia	0	3 (37.5)
Thrombocytopenia	0	1 (12.5)
Cardiac arrest	0	1 (12.5)
Colitis	0	1 (12.5)
Fatigue	0	1 (12.5)
Multiple organ dysfunction syndrome	0	1 (12.5)
Neutrophil count decreased	2 (100.0)	0
Platelet count decreased	1 (50.0)	0

DLTs were reported at 35 mg twice daily and 50 mg twice daily in part 1A only. No DLTs were reported for part 1B or 1C. No DLTs were reported at the monotherapy RDE in the single-agent or combination groups. A patient was classified as DLT-evaluable if they met the following criteria: (i) reported AE DLT on the AE page in the first 28 days from cycle 1 day 1 and (ii) received ≥75% and <110% of PF-06873600 in the first 28 days from cycle 1 day 1. The MedDRA version 25.1 coding dictionary was applied.

Abbreviations: AE, adverse event; DLT, dose-limiting toxicity; MedDRA, Medical Dictionary for Regulatory Activities; RDE, recommended dose for expansion.

### PK

**Figure 2.**
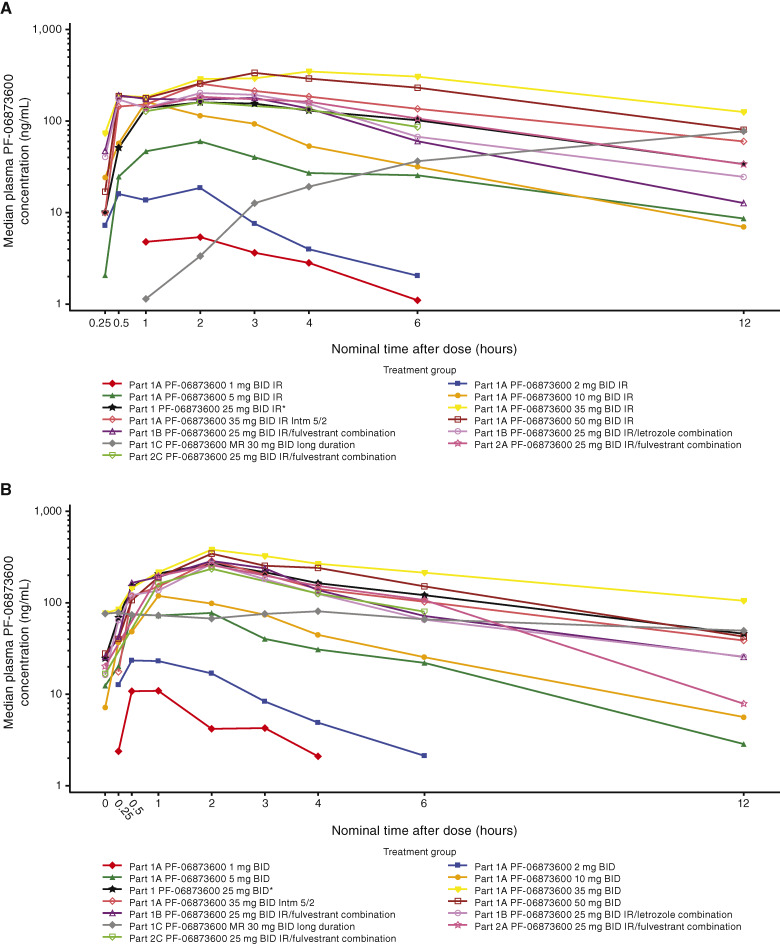
Median plasma PF-06873600 concentration–time profile. **A,** Following single oral doses on cycle 1 day 1. **B,** Following multiple oral doses on cycle 1 day 15. *Part 1 PF-06873600 25 mg twice daily (BID) IR combined with part 1A PF-06873600 25 mg BID IR, part 1A PF-06873600 25 mg BID biomarker, and part 1C PF-06873600 MR 20 mg lead-in IR 25 mg BID. IR, immediate release; MR, modified release.

Median plasma PF-06873600 concentration–time profiles following single oral doses on cycle 1 day 1 and multiple oral doses on cycle 1 day 15 are presented in Fig. 2A and B, respectively. Following oral administration of the IR formulation, PF-06873600 was rapidly absorbed. The geometric mean PK parameters of PF-06873600, including C_max_, C_min_, and AUC, generally increased with dose up to at least 35 mg twice daily.

There was minimal accumulation following repeated twice-daily dosing, with the geometric mean observed accumulation ratio (R_ac_) ranging from 0.7 to 1.3. The arithmetic mean t_½_ ranged from 2 to 3 hours. PF-06873600 exposure exhibited moderate-to-high interpatient variability. At 25 mg twice daily, the geometric mean coefficient of variation percentage values were 58% and 56% for single-dose AUC_inf_ and C_max_, respectively. After multiple dosing at 25 mg twice daily, the geometric mean coefficient of variation percentage values were 45%, 43%, and 119% for AUC_tau_, C_max_, and C_min_, respectively (Supplementary Table S3). PF-06873600 plasma exposures were largely comparable between monotherapy and combination therapy with fulvestrant or letrozole.

Two MR formulations with different release rates were evaluated in the part 1C MR selection cohort via a lead-in design (day −7 MR short duration and day −4 MR long duration). The MR long duration formulation was further evaluated with a twice-daily regimen. PK parameters of the MR formulations are summarized in Supplementary Table S4.

### Pharmacodynamics in tumor

Paired tumor biopsies at screening and cycle 2 day 1 were collected from eight patients with HR^+^/HER2^−^ mBC treated with 25 mg twice daily PF-06873600 monotherapy (part 1A) or in combination with fulvestrant (parts 1B and 2A) and analyzed for PD biomarker (pRb and Ki67 expression) changes by IHC. Following PF-06873600 IR 25 mg twice daily monotherapy, the median percentage change in Ki67-positive cells at cycle 2 day 1 from baseline was –11.7% (25th and 75th percentile range: −56.3%, 2.3%; Supplementary Fig. S1A), and the median change in pRb H-score was –16.5% (25th and 75th percentile range: −86.5%, 31.3%; Supplementary Fig. S1B). Following PF-06873600 IR 25 mg twice daily/fulvestrant combination, the median percentage change in Ki67-positive cells was –35.9% (25th–75th percentile range: −58.9%, −1.2%; Supplementary Fig. S1A), and the median percentage change in pRb H-score was –31.9% (25th–75th percentile range: −53.2%, 25.0%; Supplementary Fig. S1B).

### ctDNA molecular response

ctDNA samples from parts 2A and 2C were analyzed to evaluate the effects of PF-06873600 plus fulvestrant on ctDNA response, as measured by meanVAF changes at cycle 1 day 15 from cycle 1 day 1 and at EOT from cycle 1 day 1. At cycle 1 day 15, PF-06873600 plus fulvestrant consistently led to a significant decrease in ctDNA levels in molecular response-evaluable patients (28 in part 2A and 14 in part 2C), with comparable median decreases in meanVAF of −61% (25th–75th percentile range: −91%, −40%) and −54% (25th−75th percentile range: −95%, −16%) in parts 2A and 2C, respectively (Supplementary Fig. S2A and S2B). At EOT, ctDNA increased back to the baseline level when patients discontinued. The median ctDNA molecular response in combined parts 2A and 2C changed from −60% at cycle 1 day 15 to 6% at EOT (Supplementary Fig. S2C). Using the median cycle 1 day 15 ctDNA change (−61%) as a cutoff, patients with greater ctDNA reductions in part 2A had longer PFS compared with those with smaller reductions (HR = 0.138, 95% CI, 0.036–0.529; Supplementary Fig. S3A). A trend of improved PFS was observed for patients with greater ctDNA reduction at cycle 1 day 15 in part 2C (HR = 0.326; 95% CI, 0.034–3.15) although the sample size was small (Supplementary Fig. S3B).

### Antitumor activity

In part 1 dose escalation, partial responses (PR) were recorded for 3 of 78 patients (3.8%): part 1A (PF-06873600 IR 50 mg twice daily, mBC after CDK4/6i), part 1B (PF-06873600 IR 25 mg twice daily + fulvestrant, mBC after CDK4/6i), and part 1C (PF-06873600 IR 25 mg twice daily after two 20 mg MR lead-in doses, mBC after CDK4/6i).

In part 2A (PF-06873600 25 mg twice daily + fulvestrant, HR^+^/HER2^−^ mBC after CDK4/6i), PRs were recorded for three patients in the response-evaluable set (ORR = 6.7%, 95% CI, 1.4%–18.3%). In part 2C (PF-06873600 25 mg twice daily + fulvestrant, HR^+^/HER2^−^ mBC, CDK4/6i-naïve/after ET), PRs were recorded for five patients in the response-evaluable set (ORR = 22.7%; 95% CI, 7.8%–45.4%). For patients with HR^+^/HER2^−^ mBC with measurable disease at baseline and prior CDK4/6i (*n* = 40), a PR was observed for three patients (7.5%). Clinical benefit response [complete response (CR) + PR + stable disease of 24 weeks or more] was observed for 19 patients (42.2%) in part 2A and for 13 patients (59.1%) in part 2C (Table 4). Waterfall plots for the best percentage change from baseline in the sum of diameters for target lesions for the full analysis set are shown in Supplementary Fig. S4A for part 2A and Supplementary Fig. S4B for part 2C.

**Table 4.** Summary of best overall response and objective response (confirmed) based on investigator assessment (RECIST version 1.1)—response-evaluable set, part 2.

	Part 2A PF-06873600	Part 2C PF-06873600	Total (*N* = 67)
	25 mg twice daily IR +	25 mg twice daily IR +	
	Fulvestrant (*n* = 45)	Fulvestrant (*n* = 22)	
Confirmed best overall response, *n* (%)			
CR	0	0	0
PR	3 (6.7)	5 (22.7)	8 (11.9)
Stable disease	30 (66.7)	13 (59.1)	43 (64.2)
Non-CR/non-PD	0	1 (4.5)	1 (1.5)
Progressive disease (PD)	11 (24.4)	2 (9.1)	13 (19.4)
NE	1 (2.2)	1 (4.5)	2 (3.0)
Objective response (CR + PR), *n* (%)	3 (6.7)	5 (22.7)	8 (11.9)
95% CI^a^	1.4–18.3	7.8–45.4	5.3–22.2
Pts with HR^+^/HER2^−^ breast cancer with measurable disease at baseline	44	20	64
Objective response (pts with HR^+^/HER2^−^ breast cancer with measurable disease at baseline), *n* (%)^b^	3 (6.8)	5 (25.0)	8 (12.5)
95% CI^a^	1.4–18.7	8.7–49.1	5.6–23.2
Pts with HR^+^/HER2^−^ breast cancer with measurable disease at baseline and prior CDK4/6i	40	0	40
Objective response (CR + PR; pts with HR^+^/HER2^−^ breast cancer with measurable disease at baseline and prior CDK4/6i), *n* (%)^c^	3 (7.5)	0	3 (7.5)
95% CI^a^	1.6–20.4	NE–NE	1.6–20.4
Disease control (CR + PR + stable disease + non-CR/non-PD), *n* (%)	33 (73.3)	19 (86.4)	52 (77.6)
95% CI^a^	58.1–85.4	65.1–97.1	65.8–86.9
Clinical benefit response (CR, PR, or ≥24 weeks stable disease), *n* (%)	19 (42.2)	13 (59.1)	32 (47.8)
95% CI^a^	27.7–57.8	36.4–79.3	35.4–60.3

All values are *n* (%) unless otherwise stated.

Abbreviations: CDK4/6i, cyclin-dependent kinase 4/6 inhibitor; CI, confidence interval; CR, complete response; HER, human epidermal growth factor receptor; HR, hormone receptor; IR, immediate release; NE, not evaluable; PR, partial response; Pt, patient; RECIST, Response Evaluation Criteria in Solid Tumors.

a Clopper–Pearson method used.

b Percentages calculated out of the total number of patients with HR^+^/HER2^−^ breast cancer (with measurable disease at baseline).

c Percentages calculated out of the total number of patients with HR^+^/HER2^−^ breast cancer with measurable disease at baseline and prior CDK4/6i (palbociclib, ribociclib, and abemaciclib).

The DoR for one confirmed responder in part 2C was 5.8 months. The other seven confirmed responders were censored: one patient in part 2A for starting a new anticancer therapy, three (two in part 2A and one in part 2C) because of study termination by the sponsor, and three in part 2C for being event-free as of the cutoff date.

In part 2A (PF-06873600 + fulvestrant, HR^+^/HER2^−^ mBC after CDK4/6i), among 45 patients in the full analysis set, 27 (60%) had an event of disease progression. The estimated median PFS was 5.6 months (95% CI, 3.9–7.8). In part 2C (PF-06873600 + fulvestrant, HR^+^/HER2^−^ mBC CDK4/6i-naïve), among 28 patients in the full analysis set, there were seven events of disease progression (25.0%) and one death (3.6%). The estimated median PFS was 11.1 months (95% CI, 7.5 to not evaluable). The median (95% CI) duration of follow-up in part 2 was 10.5 (8.1, 11.1) months.

## Discussion

This phase I/IIa study evaluated the safety, PK, pharmacodynamics, and preliminary antitumor activity of the first-in-class CDK2/4/6i PF-06873600, alone and in combination with ET, in patients with HR^+^/HER2^−^ advanced breast cancer or mBC. The study included patients treated in two expansion cohorts who were CDK4/6i-naïve but had prior ET in the advanced/metastatic setting or had failed prior combination CDK4/6i and ET.

The most frequently reported TEAEs of all grades across all dose levels, as well as the most frequently observed grade 3 and grade 4 TEAEs, SAEs, and AEs leading to dose reduction and discontinuation, were hematologic (neutropenia and anemia) and GI AEs (nausea and vomiting). These findings align with the known on-target class effects of CDK4/6is. When evaluated at doses ranging from 1 to 50 mg twice daily, PF-06873600 exposures generally increased with dose up to at least 35 mg twice daily, corresponding with an increased frequency of grade 3 and grade 4 TEAEs related to hematologic and GI systems. As expected, most DLTs were also hematologic and GI in nature. An alternative intermittent dosing regimen (5 days on/2 days off) was explored at 35 mg twice daily to find the optimal balance between safety and efficacy for RDE selection. This design was supported by an interim semimechanistic neutropenia model simulation based on draft dose escalation data available for continuous regimens. The simulation predicted that this intermittent dosing regimen with equal total dose intensity as the 25 mg twice daily continuous regimen would lead to slightly higher grade 3/4 neutropenia rates while maintaining longer durations of exposure above predicted effective concentrations (C_eff_) from HCC1428, HCC1806, and Ovar3 models than the 25 mg twice daily continuous regimen. However, the clinical tolerability data were worse than the prediction.

Regarding CDK2 inhibition by PF-06873600, this is less likely to contribute to GI toxicity and may instead be more ascribed to off-target CDK activities, potentially stemming from inhibition of CDK1. Although CDK2 is an important cell-cycle kinase, CDK2 knockout mice remain viable without exhibiting GI phenotypes (10). An *in vitro* study involving selective knockdown of individual CDKs (CDK1, CDK2, CDK4, CDK6, and CDK9) in rat intestinal epithelial cells revealed that only the loss of CDK9 and CDK1, but not CDK2, CDK4, or CDK6, led to significant inhibition of cell proliferation and induction of apoptosis. This underscores the essential role of CDK1 in intestinal epithelial cell homeostasis (11). Notably, PF-06873600 exhibits only modest biochemical selectivity for CDK1 (Ki = 4.5 nmol/L) compared with its main target, CDK4 (Ki = 1.25 nmol/L), in contrast to the selectivity for CDK9 (Ki = 19.6 nmol/L; ref. 7). Therefore, further enhancing CDK1 selectivity may help improve GI tolerability.

The underlying factors contributing to the observed moderate-to-high PK variability are not fully understood. With dose reductions due to AEs, most patients were at their maximum tolerable dose after 1 to 2 cycles of study drug treatment. Food effect evaluation was not performed with this molecule in the clinical setting. Double absorption peaks were observed in some patients, but it is not clear if food intake outside the fasting window contributed to these exposure profiles. Mean steady-state PF-06873600 PK concentrations at the RDE of 25 mg twice daily were maintained above the predicted effective concentrations (C_eff_) from HCC1428, HCC1806, and Ovar3 models for at least 70% of the dosing interval, but this criterion was not achieved for C_eff_ from the MCF7 model.

Consistent with its mechanism of action, PF-06873600 treatment led to modest inhibition of CDK PD biomarkers pRb and Ki67 in tumor biopsy tissues that was further enhanced by the combination with fulvestrant, indicating target engagement and modulation. However, relatively high interpatient variability was observed, as reflected by the respective 25th to 75th percentile range values. This may be attributed to the moderate-to-high interpatient PK variability and the short half-life of PF-06873600, which, combined with the low therapeutic index at the achievable RDE, can result in suboptimal depth and duration of target coverage and inhibition in some patients. The observed Ki67 inhibition achieved by single-agent PF-06873600 was less than that observed for CDK4/6i ribociclib (mean inhibition ∼60%–70% at 600 mg daily) although the latter was measured in multiple tumor types (12). Stronger pRb and Ki67 inhibition may be needed at higher dose levels of PF-06873600 to achieve the required target coverage for full clinical activity.

An early decrease of ctDNA after 14 days of treatment with PF-06873600 plus fulvestrant was observed in patients with mBC, both with and without prior CDK4/6i treatment. This inhibition was lost, and ctDNA increased to baseline levels at EOT when patients developed progressive disease. Plasma ctDNA levels can reflect tumor burden (13). In HR^+^/HER2^−^ mBC, treatment with CDK4/6i plus ET led to an early decrease of ctDNA, or molecular response, which can be both a pharmacodynamic biomarker and predictive of a reduced risk of disease progression (14, 15). Consistent with this, patients with greater ctDNA reduction at cycle 1 day 15 were more likely to have longer PFS. These results indicate that PF-06873600 had an impact on tumor cell growth and DNA shedding, and stronger ctDNA effects were more likely to lead to favorable clinical outcomes. However, these findings were from a small number of patients and need to be further evaluated in a larger population.

As monotherapy, PF-06873600 demonstrated modest antitumor activity in HR^+^/HER2^−^ mBC, with one PR at the 50 mg twice daily dose level and one PR at 20 mg MR/25 mg twice daily. In dose expansion cohorts, PF-06873600 combined with fulvestrant showed promising early efficacy. In part 2A (patients with HR^+^/HER2^−^ mBC who progressed after prior CDK4/6i treatment), the ORR was 6.7% (3/45), CBR (CR + PR + stable disease ≥ 24 weeks) was 42.2% (19/45), and the disease control rate (CR + PR + stable disease + non-CR/non-PD) was 73.3% (33/45). In part 2C (patients with HR^+^/HER2^−^ mBC and CDK4/6i-naïve who progressed on prior ET), the ORR was 22.7% (5/22), CBR was 59.1% (13/22), and the disease control rate was 86.4% (19/22). The estimated median PFS was 5.6 months (95% CI, 3.9–7.8) in part 2A and 11.1 months (95% CI, 7.5 to not evaluable) in part 2C. The results in the post-ET patient population were comparable with the pivotal PALOMA3 study of palbociclib and fulvestrant, which reported a median PFS of 9.5 months (95% CI, 9.2–11.0) and an ORR of 24.6% (95% CI, 19.6–30.2; refs. 16, 17).

Patients with mBC who receive CDK4/6is eventually develop resistance. This unmet need drives research into novel approaches to enhance treatment efficacy and delay resistance. The biological rationale behind combining CDK4 and CDK2 inhibitors is multifaceted. CDK4/6is primarily target the G_1_–S checkpoint by inhibiting CDK4 and CDK6, whereas CDK2 inhibitors also promote cell-cycle arrest. By simultaneously inhibiting both CDK4/6 and CDK2, this approach maximizes tumor growth inhibition. However, challenges exist. Overlapping toxicities complicate dosing decisions, and balancing specificity (targeting the right kinases) with efficacy is crucial. Next-generation inhibitors, such as CDK4- and CDK2-selective compounds, may allow titration of each component separately, optimizing the therapeutic window. In summary, PF-06873600 showed clinical activity and was well tolerated at the 25 mg twice-daily dose; however, Pfizer discontinued further development to focus on the development of the CDK4- and CDK2-selective inhibitors.

Ongoing clinical trials are currently assessing novel combinations such as PF-07104091 (CDK2-selective inhibitor) with PF-07220060 (CDK4-selective inhibitor) plus ET in advanced HR^+^/HER2^−^ breast cancer (NCT05262400). Additionally, the orally available RGT419B selectively targets CDK4 over CDK2 or CDK6, offering promise for addressing resistance (18). In summary, understanding the intricate interplay among CDK4, CDK6, and CDK2 provides exciting avenues for improving mBC treatment.

## Supplementary Material

Supplementary Table S1 Supplementary Table S1. Representativeness of study participants.

Supplementary Table S2 Supplementary Table S2. Summary of treatment-emergent adverse events (>15%) by preferred term and maximum CTCAE grade (treatment-related, all cycles)—safety analysis set, Part 2.

Supplementary Table S3 Supplementary Table S3. Descriptive summary of plasma PF-06873600 immediate release formulation PK parameters—PK parameter analysis set.

Supplementary Table S4 Supplementary Table S4. Descriptive summary of plasma PF-06873600 PK parameters following the immediate release, modified release short duration, and modified release long duration formulations (Part 1C)—PK parameter analysis set.

Supplementary Figure S1 Supplementary Figure S1. Change from baseline in tumor biomarkers following monotherapy or combination treatment - biomarker analysis set.

Supplementary Figure S2 Supplementary Figure S2. ctDNA molecular response.

Supplementary Figure S3 Supplementary Figure S3. Median ctDNA change and PFS.

Supplementary Figure S4 Supplementary Figure S4. Waterfall plot for best percent change from baseline in sum of diameters for target lesions based on investigator assessment (RECIST v1.1) - full analysis set, Part 2.
