# Supplementary material for: First-in-Human Phase I/IIa Study of the First-in-Class CDK2/4/6 Inhibitor PF-06873600 Alone or with Endocrine Therapy in Patients with Breast Cancer
Source: Clin Cancer Res. 2025 Apr 17;31(14):2899–909. doi: 10.1158/1078-0432.CCR-24-2740 (PMC12260505; doi:10.1158/1078-0432.CCR-24-2740)
Supplement: Supplementary Table S1 — Representativeness of study participants. [file ccr-24-2740_supplementary_table_s1_suppst1.pdf]

Supplementary Table S1. Representativeness of study participants

Cancer type	HR+/HER2– advanced or mBC
Considerations related to:	
Sex	HR+/HER2– advanced or mBC, as well as all other subtypes of breast cancer, is a predominantly female disease and is rare in men. Less than 1% of all breast cancer occurs in men.
Age	The median age at diagnosis is around 62 years for all breast cancer.
Race/ethnicity	In the United States, the age-adjusted rate of new female HR+/HER2– breast cancer is 100.8 per 100,000 in White patients and 76.1 per 100,000 in Black patients.
Geography	This study was conducted in 5 countries (United States, Japan, Ukraine, Bulgaria, and Canada) with the majority of treated patients (79.5%) in the United States.<br><br>In the United States, there were approximately 300,590 new cases of breast cancer in 2023. In Japan, there were approximately 97,300 new cases of breast cancer in 2023. In Ukraine, there were approximately 18,096 new cases of breast cancer in 2022. In Bulgaria, there were approximately 3558 new cases of breast cancer in 2022. In Canada, there were approximately 29,400 new cases of breast cancer in 2023.
Other considerations	Although the rates of new cases of HR+/HER2− breast cancer is lower in Black women than White women, the 5-year survival rate for Black women is lower at 88% vs 96% for White women.
Overall representativeness	The median age in this study (59 years) was similar to the reported median age at diagnosis for breast cancer (62 years). Black women were underrepresented in our study, only making up 6.6% of the study population. Only women were included in this study which is consistent with rarity of male breast cancer.

HER2−, human epidermal growth factor receptor–negative; HR+, hormone receptor–positive; mBC, metastatic breast cancer.
