# Supplementary material for: First-in-Human Phase I/IIa Study of the First-in-Class CDK2/4/6 Inhibitor PF-06873600 Alone or with Endocrine Therapy in Patients with Breast Cancer
Source: Clin Cancer Res. 2025 Apr 17;31(14):2899–909. doi: 10.1158/1078-0432.CCR-24-2740 (PMC12260505; doi:10.1158/1078-0432.CCR-24-2740)
Supplement: Supplementary Table S2 — Summary of treatment-emergent adverse events (>15%) by preferred term and maximum CTCAE grade (treatment-related, all cycles)—safety analysis set, Part 2. [file ccr-24-2740_supplementary_table_s2_suppst2.pdf]

Supplementary Table S2. Summary of treatment-emergent adverse events (>15%) by preferred term and maximum CTCAE grade (treatment-related, all cycles)—safety analysis set, Part 2.

	**Part 2A PF-06873600 25 mg BID IR + Fulvestrant (*N* = 45)**					**Part 2C PF-06873600 25 mg BID IR + Fulvestrant (*N* = 28)**				
**Preferred term**	**Grade 1**	**Grade 2**	**Grade 3/4**	**Grade 5**	**Total**	**Grade 1**	**Grade 2**	**Grade 3/4**	**Grade 5**	**Total**
Any adverse event	9 (20.0)	17 (37.8)	19 (42.2)	0	45 (100.0)	7 (25.0)	8 (28.6)	8 (28.6)	1 (3.6)	24 (85.7)
Nausea	22 (48.9)	6 (13.3)	1 (2.2)	0	29 (64.4)	12 (42.9)	7 (25.0)	0	0	19 (67.9)
Fatigue	10 (22.2)	13 (28.9)	5 (11.1)	0	28 (62.2)	7 (25.0)	0	2 (7.1)	0	9 (32.1)
Neutropenia	4 (8.9)	5 (11.1)	13 (28.9)	0	22 (48.9)	1 (3.6)	2 (7.1)	6 (21.4)	0	9 (32.1)
Anemia	3 (6.7)	10 (22.2)	7 (15.6)	0	20 (44.4)	4 (14.3)	3 (10.7)	3 (10.7)	0	10 (35.7)
Alopecia	7 (15.6)	9 (20.0)	0	0	16 (35.6)	6 (21.4)	2 (7.1)	0	0	8 (28.6)
Headache	12 (26.7)	4 (8.9)	0	0	16 (35.6)	9 (32.1)	0	0	0	9 (32.1)
Vomiting	12 (26.7)	3 (6.7)	1 (2.2)	0	16 (35.6)	8 (28.6)	4 (14.3)	0	0	12 (42.9)
Leukopenia	1 (2.2)	8 (17.8)	2 (4.4)	0	11 (24.4)	3 (10.7)	3 (10.7)	0	0	6 (21.4)
Diarrhea	7 (15.6)	1 (2.2)	2 (4.4)	0	10 (22.2)	4 (14.3)	1 (3.6)	0	0	5 (17.9)
Thrombocytopenia	5 (11.1)	2 (4.4)	3 (6.7)	0	10 (22.2)	1 (3.6)	0	1 (3.6)	0	2 (7.1)
Constipation	4 (8.9)	5 (11.1)	0	0	9 (20.0)	1 (3.6)	2 (7.1)	0	0	3 (10.7)
Abdominal pain	7 (15.6)	1 (2.2)	0	0	8 (17.8)	0	0	0	0	0
Dizziness	6 (13.3)	2 (4.4)	0	0	8 (17.8)	0	1 (3.6)	0	0	1 (3.6)
Decreased appetite	6 (13.3)	1 (2.2)	0	0	7 (15.6)	2 (7.1)	0	0	0	2 (7.1)

All values are *n* (%). Treatment-emergent adverse events occurring in >15% of patients in Part 2A or Part 2C shown.

BID, twice daily; CTCAE, Common Terminology Criteria for Adverse Events; IR, immediate release.
