# Supplementary material for: First-in-Human Phase I/IIa Study of the First-in-Class CDK2/4/6 Inhibitor PF-06873600 Alone or with Endocrine Therapy in Patients with Breast Cancer
Source: Clin Cancer Res. 2025 Apr 17;31(14):2899–909. doi: 10.1158/1078-0432.CCR-24-2740 (PMC12260505; doi:10.1158/1078-0432.CCR-24-2740)
Supplement: Supplementary Table S3 — Descriptive summary of plasma PF-06873600 immediate release formulation PK parameters—PK parameter analysis set. [file ccr-24-2740_supplementary_table_s3_suppst3.pdf]

Supplementary Table S3. Descriptive summary of plasma PF-06873600 immediate release formulation PK parameters—PK parameter analysis set.

Parameter (unit)	Part 1A									Part 1B		Part 1C	Part 1
	1 mg BID	2 mg BID	5 mg BID	10 mg BID	25 mg BID	25 mg BID Biomarker	35 mg BID	35 mg BID Intm 5/2	50 mg BID	25 mg BID +Ful	25 mg BID +Let	MR 20 mg lead-in IR 25 mg BID	25 mg BID[b]
**Cycle1/Day 1**													
N1, N2[a]	1, 1	2, 1	2, 1	3, 3	9, 6	7, 5	4, 2	13, 9	10, 4	9, 6	6, 6	5, 3	21, 14
AUC_{inf} (ng.h/mL)	21.0	45.2	101	648.4 (60)	1492 (20)	892.6 (73)	2010, 3400	1614 (48)	1875 (41)	884.3 (34)	1142 (50)	593.3 (17)	1019 (58)
AUC_{last} (ng.h/mL)	18.3	39.2, 58.3	96.6, 442	599.4 (53)	1295 (25)	787.9 (59)	2510 (25)	1395 (38)	1976 (49)	1034 (43)	1040 (46)	596.8 (33)	912.4 (52)
CL/F (L/h)	47.6	44.2	49.7	15.43 (60)	16.78 (20)	28.04 (73)	10.3, 17.4	21.70 (48)	26.63 (41)	28.26 (34)	21.87 (50)	42.14 (17)	24.55 (58)
C_{max} (mg/mL)	5.39	22.1, 31.9	29.1, 90.8	164.1 (37)	243.5 (47)	202.6 (47)	388.1 (9)	276.4 (42)	393.8 (42)	225.4 (28)	228.5 (32)	121.8 (54)	194.2 (56)
$t_{1/2}$ (h)	1.61	1.82	1.96	2.567± 0.82033	2.540± 0.14394	2.446± 0.54574	2.39, 3.74	2.809± 0.59669	2.163± 0.74732	2.128± 0.73966	2.428± 0.87316	2.250± 0.77350	2.444±0.45232
T_{max} (h)	2.17 (2.17–2.17)	1.25 (0.50–2.00)	2.02 (2.00–2.03)	0.983 (0.50–1.90)	2.17 (1.10–5.47)	1.90 (0.517–3.00)	3.64 (2.00–5.92)	2.02 (0.517–6.00)	3.49 (0.55–6.03)	1.97 (0.533–5.95)	2.52 (0.50–3.00)	2.00 (0.517–10.3)	2.00 (0.517–10.3)
V_z/F (L)	111	116	141	55.32 (28)	61.33 (20)	97.32 (56)	55.5, 60.0	86.06 (37)	79.74 (15)	83.54 (17)	72.18 (37)	132.2 (52)	85.26 (52)
**Cycle 1/Day 15**													
N1, N2[a]	1, 1	2, 2	2, 2	3, 3	9, 9	7, 7	2, 2	9, 7	6, 4	8, 7	7, 7	NA	16, 16
AUC_{tau} (ng.h/mL)	25.3	51.4, 86.9	150, 483	448.8 (25)	1695 (34)	1010 (40)	2060, 2360	1372 (39)	1607 (34)	1229 (41)	1273 (50)	NA	1351 (45)
CL/F (L/h)	39.5	23.0, 38.9	10.4, 33.2	22.28 (24)	14.78 (34)	24.76 (40)	14.8, 17.0	25.54 (39)	21.99 (44)	20.35 (41)	19.63 (49)	NA	18.53 (45)
C_{max} (ng/mL)	10.9	18.2, 45.4	46.3, 126	124.3 (29)	382.4 (33)	246.4 (40)	362, 399	290.2 (29)	335.2 (22)	305.0 (35)	276.5 (31)	NA	315.5 (43)
C_{min} (ng/mL)	0.000	0.000, 0.000	1.19, 23.6	5.499 (5)	37.63 (112)	21.28 (130)	52.1, 80.4	25.56 (8)	25.63 (101)	21.25 (106)	13.33 (204)	NA	30.23 (119)
R_{ac}	1.21	1.16	1.53	0.7263 (28)	1.045 (17)	1.254 (49)	0.822	0.8893 (32)	1.260 (25)	1.191 (29)	1.268 (35)	NA	1.135 (34)
$t_{1/2}$ (h)	–	1.61, 1.75	2.59	2.250± 0.21517	2.733± 0.49286	2.797± 0.70053	3.31	2.752± 0.68727	2.535± 1.0967	2.508± 0.84281	2.468± 0.90126	NA	2.765± 0.57842

T_{max} (h)	1.00 (1.00–1.00)	1.25 (0.50–2.00)	1.20 (0.50–1.90)	2.00 (0.25–2.13)	1.95 (0.90–5.70)	1.92 (1.00–2.92)	2.01 (2.00–2.02)	2.23 (0.00–4.05)	1.98 (1.10–4.25)	1.88 (0.517–2.12)	2.08 (1.82–3.93)	NA	1.94 (0.90–5.70)
V_z/F (L)	–	53.3, 98.4	124	72.17 (19)	57.62 (29)	99.62 (35)	70.8	99.03 (48)	75.41 (17)	75.71 (32)	80.33 (27)	NA	75.77 (43)

Data are geometric mean (geometric % coefficient of variation) for all except median (range) for Tmax and arithmetic mean±standard deviation for t1/2. Standard deviation, geometric CV (%) are not presented if the number of evaluable measurements for a parameter <3. Individual values are listed if the number of evaluable measurements for a parameter <3.

[a] N1 is number of patients contributing to the summary statistics of each visit; N2 is number of patients contributing to the summary statistics for AUCinf, CL/F, t1/2, and Vz/F of each visit.

[b] Part 1 (Cycle 1 Day 1) PF-06873600 25 mg BID combined Part 1A PF-06873600 25 mg BID, Part 1A PF-06873600 25 mg BID Biomarker, and Part 1C PF-06873600 MR 20 mg IR 25 mg BID. Part 1 (Cycle 1 Day 15) PF-06873600 25 mg BID combined Part 1A PF-06873600 25 mg BID and Part 1A PF-06873600 25 mg BID Biomarker.

AUC_{inf}, area under the concentration-time profile from time 0 to infinite time; AUC^{last}, area under the concentration-time profile from 0 to time of last quantifiable concentration; BID, twice daily; CL/F, apparent clearance; C_{max}, maximum plasma concentration; C_{min}, lowest concentration observed during the dosing interval; NA, not applicable; PK, pharmacokinetics; R_{ac}, observed accumulation ratio based on AUC ; $t_{1/2}$, terminal phase half-life; T_{max}, time to reach C_{max} ; V_z/F, apparent volume of distribution
