# Supplementary material for: First-in-Human Phase I/IIa Study of the First-in-Class CDK2/4/6 Inhibitor PF-06873600 Alone or with Endocrine Therapy in Patients with Breast Cancer
Source: Clin Cancer Res. 2025 Apr 17;31(14):2899–909. doi: 10.1158/1078-0432.CCR-24-2740 (PMC12260505; doi:10.1158/1078-0432.CCR-24-2740)
Supplement: Supplementary Table S4 — Descriptive summary of plasma PF-06873600 PK parameters following the immediate release, modified release short duration, and modified release long duration formulations (Part 1C)—PK parameter analysis set. [file ccr-24-2740_supplementary_table_s4_suppst4.pdf]

Supplementary Table S4. Descriptive summary of plasma PF-06873600 PK parameters following the immediate release, modified release short duration, and modified release long duration formulations (Part 1C)—PK parameter analysis set.

Parameter (unit)	MR 20 mg IR lead-in 25 mg BID	MR 30 mg BID long duration
**Day -7**		
N1, N2[a]	5, 5	NA
AUC_{inf} (ng.h/mL)	687.8 (73)	NA
AUC_{last} (ng.h/mL)	639.7 (67)	NA
CL/F (L/h)	29.05 (73)	NA
C_{max} (mg/mL)	73.72 (58)	NA
$t_{1/2}$ (h)	6.778±5.7890	NA
T_{max} (h)	5.33 (2.00–6.00)	NA
V_z/F (L)	218.1 (50)	NA
**Day -4**		
N1, N2[a]	5, 2	NA
AUC_{inf} (ng.h/mL)	584, 713	NA
AUC_{last} (ng.h/mL)	469.7 (63)	NA
CL/F (L/h)	28.0, 34.3	NA
C_{max} (mg/mL)	39.03 (71)	NA
$t_{1/2}$ (h)	4.03, 10.0	NA
T_{max} (h)	12.0 (6.00–12.0)	NA
V_z/F (L)	163, 496	NA
**Cycle 1/Day 1**		
N1, N2[a]	5, 3	6, 0
AUC_{inf} (ng.h/mL)	593.3 (17)	–
AUC_{last} (ng.h/mL)	596.8 (33)	192.0 (201)
CL/F (L/h)	42.14 (17)	–
C_{max} (mg/mL)	121.8 (54)	43.51 (121)
$t_{1/2}$ (h)	2.250±0.77350	–
T_{max} (h)	2.00 (0.517–10.3)	10.0 (5.35–10.2)
V_z/F (L)	132.2 (52)	–
**Cycle 1/ Day 15**		
N1, N2[a]	NA	6, 6
AUC_{tau} (ng.h/mL)	NA	636.8 (59)
CL/F (L/h)	NA	47.08 (59)
C_{max} (mg/mL)	NA	82.11 (72)

C_{min} (ng/mL)	NA	45.78 (49)
T_{max} (h)	NA	2.99 (0.000–4.00)

Geometric mean (geometric % coefficient of variation) for all except median (range) for T_{max} and arithmetic mean±standard deviation for $t_{1/2}$. Standard deviation, geometric CV (%) were not presented if the number of evaluable measurements for a parameter <3. Individual values were listed if the number of evaluable measurements for a parameter <3.

[a] N1 is number of patients contributing to the summary statistics of each visit; N2 is number of patients contributing to the summary statistics for AUC_{inf}, CL/F, $t_{1/2}$, and V_z/F of each visit.

IR, immediate release; MR, modified release; AUC_{inf}, area under the concentration-time profile from time 0 to infinite time; AUC_{last}, area under the concentration-time profile from 0 to time of last quantifiable concentration; AUC_{tau}, area under the concentration-time profile from time 0 to time tau (τ), the dosing interval; CL/F, apparent clearance; C_{max}, maximum plasma concentration; NA, not applicable; PK, pharmacokinetics; $t_{1/2}$, terminal phase half-life; T_{max}, time to reach C_{max} ; V_z/F, apparent volume of distribution

Single dose: Cycle 1 Day 1 for 25 mg IR (MR 20 mg IR lead-in 25 mg BID cohort) and 30 mg MR long duration; Day-7 for 20 mg MR Short Duration (MR 20 mg IR lead-in 25 mg BID cohort); Day-4 for 20 mg MR Long Duration (MR 20 mg IR lead-in 25 mg BID cohort).

Last nominal sampling time for MR 20 mg IR lead-in phase is 24 hours (±40 min) post dose. Last nominal sampling time for BID cohort is 12 hours (±120 min) post dose.
