# Supplementary material for: First-in-Human Phase I/IIa Study of the First-in-Class CDK2/4/6 Inhibitor PF-06873600 Alone or with Endocrine Therapy in Patients with Breast Cancer
Source: Clin Cancer Res. 2025 Apr 17;31(14):2899–909. doi: 10.1158/1078-0432.CCR-24-2740 (PMC12260505; doi:10.1158/1078-0432.CCR-24-2740)
Supplement: Supplementary Figure S1 — Change from baseline in tumor biomarkers following monotherapy or combination treatment - biomarker analysis set. [file ccr-24-2740_supplementary_figure_s1_suppsf1.pdf]

Supplementary Figure S1. Change from baseline in tumor biomarkers following monotherapy or combination treatment – biomarker analysis set. **A,** change in Ki67-positive cells (n=8). Lines overlap for 2 patients in combination therapy who had the same baseline to C2D1 values (30% to 35%). **B,** change in pRb H-score (n=8).

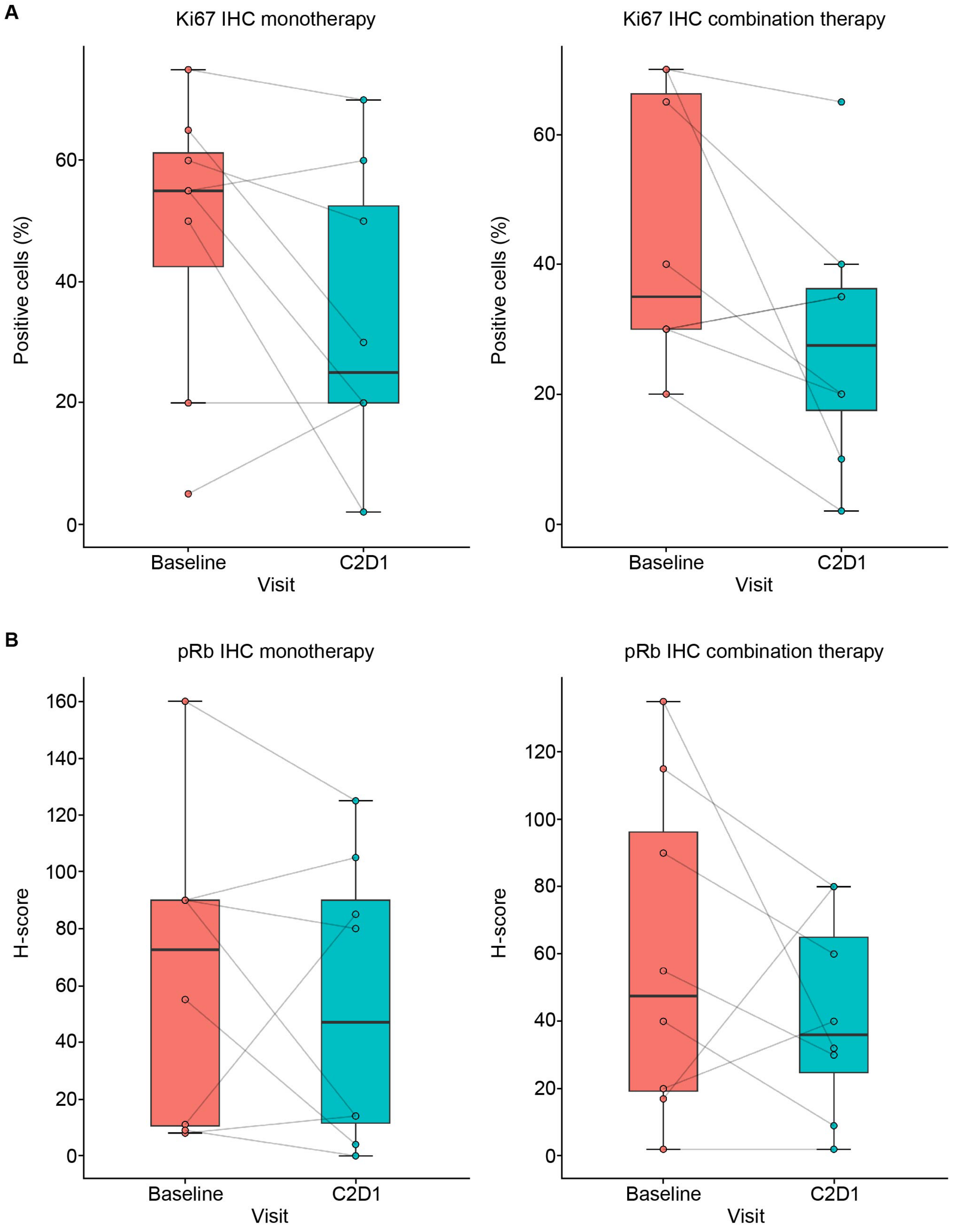

C, cycle; D, day; IHC, immunohistochemistry; pRb, phosphorylated Rb
