# Supplementary material for: First-in-Human Phase I/IIa Study of the First-in-Class CDK2/4/6 Inhibitor PF-06873600 Alone or with Endocrine Therapy in Patients with Breast Cancer
Source: Clin Cancer Res. 2025 Apr 17;31(14):2899–909. doi: 10.1158/1078-0432.CCR-24-2740 (PMC12260505; doi:10.1158/1078-0432.CCR-24-2740)
Supplement: Supplementary Figure S2 — ctDNA molecular response. [file ccr-24-2740_supplementary_figure_s2_suppsf2.pdf]

Supplementary Figure S2. ctDNA molecular response. **A,** Part 2A: C1D1 vs C1D15. **B,** Part 2C: C1D1 vs C1D15. **C,** Part 2A and 2C: C1D15 vs EOT

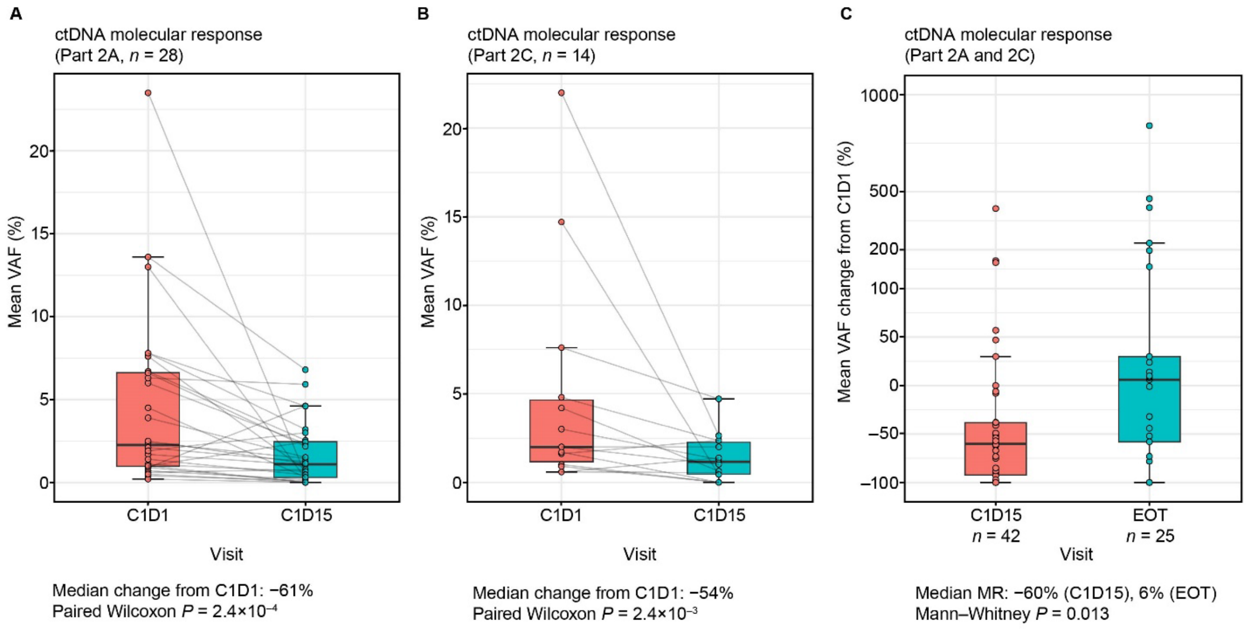

C, cycle; ctDNA, circulating tumor DNA; D, day; EOT, end of treatment; MR, molecular response; VAF, variant allele frequency
