# Supplementary material for: First-in-Human Phase I/IIa Study of the First-in-Class CDK2/4/6 Inhibitor PF-06873600 Alone or with Endocrine Therapy in Patients with Breast Cancer
Source: Clin Cancer Res. 2025 Apr 17;31(14):2899–909. doi: 10.1158/1078-0432.CCR-24-2740 (PMC12260505; doi:10.1158/1078-0432.CCR-24-2740)
Supplement: Supplementary Figure S3 — Median ctDNA change and PFS. [file ccr-24-2740_supplementary_figure_s3_suppsf3.pdf]

Supplementary Figure S3: Median ctDNA change and PFS. **A,** Part 2A. **B,** Part 2C

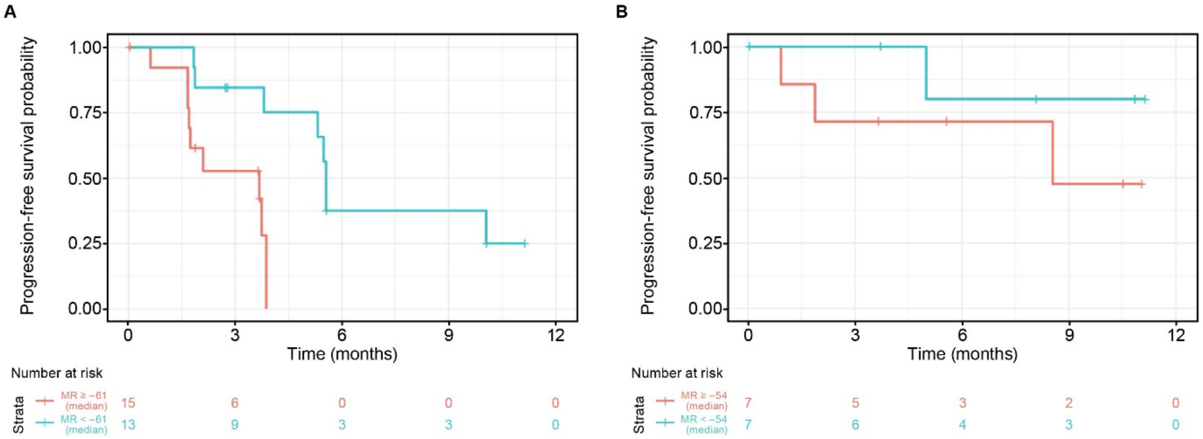

ctDNA, circulating DNA; MR, molecular response; PFS, progression-free survival
