# Supplementary material for: First-in-Human Phase I/IIa Study of the First-in-Class CDK2/4/6 Inhibitor PF-06873600 Alone or with Endocrine Therapy in Patients with Breast Cancer
Source: Clin Cancer Res. 2025 Apr 17;31(14):2899–909. doi: 10.1158/1078-0432.CCR-24-2740 (PMC12260505; doi:10.1158/1078-0432.CCR-24-2740)
Supplement: Supplementary Figure S4 — Waterfall plot for best percent change from baseline in sum of diameters for target lesions based on investigator assessment (RECIST v1.1) - full analysis set, Part 2. [file ccr-24-2740_supplementary_figure_s4_suppsf4.pdf]

Supplementary Figure S4. Waterfall plot for best percent change from baseline in sum of diameters for target lesions based on investigator assessment (RECIST v1.1) – full analysis set, Part 2. **A,** Part 2A. **B,** Part 2C

A

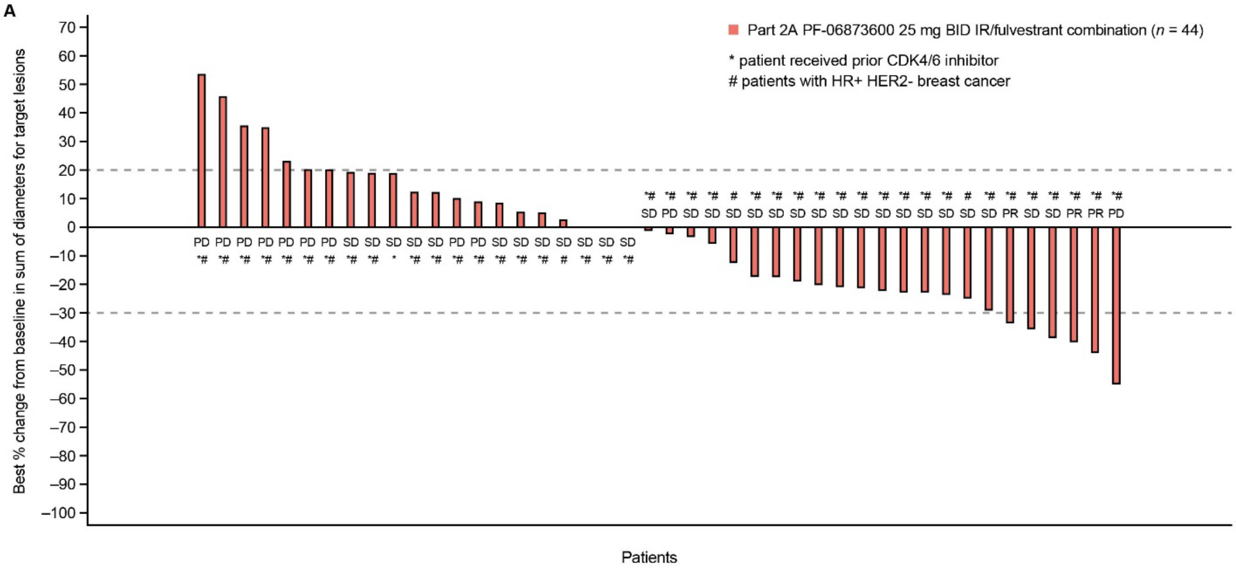

B

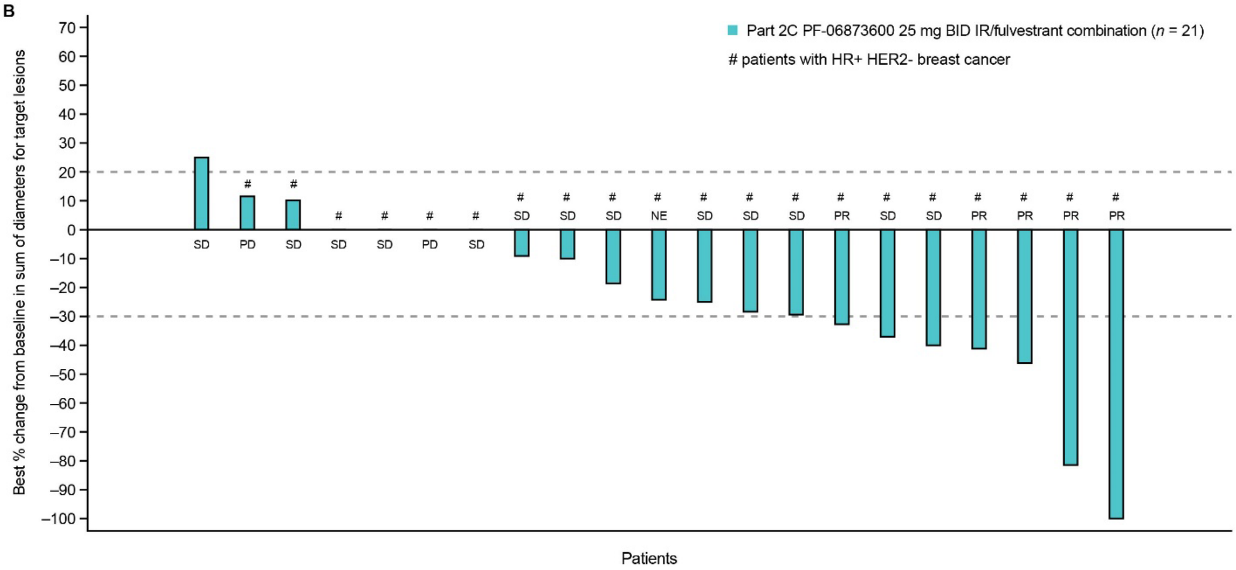

BID, twice daily; IR, immediate release; NE, not evaluable; PD, progressive disease; PR, partial response; RECIST, Response Evaluation Criteria in Solid Tumors; SD, stable disease
